# The Role of Hospital Inpatients in Supporting Medication Safety: A Qualitative Study

**DOI:** 10.1371/journal.pone.0153721

**Published:** 2016-04-19

**Authors:** Sara Garfield, Seetal Jheeta, Fran Husson, Jill Lloyd, Alex Taylor, Charles Boucher, Ann Jacklin, Anna Bischler, Christine Norton, Rob Hayles, Bryony Dean Franklin

**Affiliations:** 1 Centre for Medication Safety and Service Quality, Imperial College Healthcare NHS Trust, London, United Kingdom; 2 Research Department of Practice and Policy, UCL School of Pharmacy, Mezzanine Floor, BMA House, Tavistock Square, London, United Kingdom; 3 Pharmacy Department, Chelsea and Westminster Healthcare NHS Foundation Trust, London, United Kingdom; 4 King’s College London, Faculty of Nursing & Midwifery & Imperial College Healthcare NHS Trust, London, United Kingdom; 5 Patients' Participation Group, Walm Lane Surgery, London, United Kingdom; 6 Meeting of Minds, London, United Kingdom; 7 Collaborative Leadership in Applied Healthcare Research Northwest London, London, United Kingdom; University of Rochester, UNITED STATES

## Abstract

**Background:**

Inpatient medication errors are a significant concern. An approach not yet widely studied is to facilitate greater involvement of inpatients with their medication. At the same time, electronic prescribing is becoming increasingly prevalent in the hospital setting. In this study we aimed to explore hospital inpatients’ involvement with medication safety-related behaviours, facilitators and barriers to this involvement, and the impact of electronic prescribing.

**Methods:**

We conducted ethnographic observations and interviews in two UK hospital organisations, one with established electronic prescribing and one that changed from paper to electronic prescribing during our study. Researchers and lay volunteers observed nurses’ medication administration rounds, pharmacists’ ward rounds, doctor-led ward rounds and drug history taking. We also conducted interviews with healthcare professionals, patients and carers. Interviews were audio-recorded and transcribed. Observation notes and transcripts were coded thematically.

**Results:**

Paper or electronic medication records were shown to patients in only 4 (2%) of 247 cases. However, where they were available during patient-healthcare professional interactions, healthcare professionals often viewed them in order to inform patients about their medicines and answer any questions. Interprofessional discussions about medicines seemed more likely to happen in front of the patient where paper or electronic drug charts were available near the bedside. Patients and carers had more access to paper-based drug charts than electronic equivalents. However, interviews and observations suggest there are potentially more significant factors that affect patient involvement with their inpatient medication. These include patient and healthcare professional beliefs concerning patient involvement, the way in which healthcare professionals operate as a team, and the underlying culture.

**Conclusion:**

Patients appear to have more access to paper-based records than electronic equivalents. However, to develop interventions to increase patient involvement with medication safety behaviours, a wider range of factors needs to be considered.

## Background

Medication errors are common in hospital inpatients. UK research suggests that prescribing errors occur in up to 15% of inpatient medication orders and 9% of medications prescribed at discharge [1]. A recent meta-analysis reports medication administration errors in 5.6% of non-intravenous doses and 35% of intravenous doses in UK hospitals [2]. Although many of these do not result in patient harm, some have serious consequences, and even errors that do not cause harm can affect patients’ confidence in their healthcare.

While many interventions have been proposed to address these problems, few have been shown to have significant benefits. Furthermore, solutions shown to be helpful elsewhere may not extrapolate from one country to another due to significant international differences in how medication is prescribed, dispensed and administered [3]. A complementary approach to reducing errors, not yet widely studied, is to facilitate greater involvement of patients [4,5]. In particular, patients with long term conditions (and their carers) are likely to know a great deal about their usual medication. Patients are thus therefore a potentially important (and often the final) defense against errors relating to their medication.

Patient safety activities relating to inpatient medication may include (but are not limited to) viewing their inpatient medication records, prompting staff to avoid dose omissions, providing information to aid handover between shifts and professional groups, and raising queries with doctors, pharmacists or nursing staff. For example, in one Swiss study, the majority of oncology patients were confident that they could watch for errors and notify staff [6]. Within England, an ethnographic study of medication administration rounds has confirmed that patients do query their medication with ward staff, and sometimes prevent potential medication errors [7]. In addition, an Australian chart review study found that patients identified prescribing errors [8]. However, previous research [9] suggests that patients are often unsure of the medication they are prescribed as an inpatient, preventing effective engagement with their treatment and thus a more active role in medication safety. Research has shown that patients are more willing to participate in patient safety if encouraged to do so by healthcare professionals [10–12]. However, there is relatively little research in this area and it is not known which interventions to increase patient involvement lead to improved healthcare outcomes [13]. Further work is therefore needed to investigate the roles that healthcare professionals and patients believe are appropriate for hospital inpatients to take relating to safety [14].

In most UK hospitals, inpatients’ medication is prescribed on a paper drug chart (paper based medication record), usually kept in a folder at the end of their bed with other charts. While primarily designed for use by healthcare professionals involved with prescribing, dispensing and administering medication, the drug chart is also potentially available for the patient to look at. It has been suggested that a well written drug chart should also be clear and legible to the patient [15] although recent good practice guidance for the design of inpatient medication charts [16] does not refer to patients as potential users. However, electronic prescribing (EP) is becoming increasingly prevalent in the hospital setting both in the UK and elsewhere. In a survey of all 165 English acute hospital trusts, we found that 13% of 101 responding hospitals had EP in all surgical and medical wards [17] with many more hospitals using EP in certain clinical areas. While there are many potential benefits of EP, including a reduction in medication errors and adverse events [18], this means that instead of inpatient medication being prescribed on a paper drug chart at the end of the patient’s bed, it is prescribed on a computer or other electronic device. This digitalisation of inpatient medication records could be a barrier or facilitator to inpatients being involved with their inpatient medication. We have previously explored inpatients’ views on the introduction of such systems [19], but little is known about how EP may change the extent to which hospital inpatients are aware of their prescribed medication and feel able to actively engage in safety related activities such as querying healthcare professionals if they feel that a medication may have been prescribed, dispensed or administered (or omitted) in error.

In this study we aimed to identify how hospital inpatients engaged with medication safety-related behaviours in two UK hospital trusts, the facilitators and barriers to this engagement, and the impact of EP.

## Methods

### Ethics statement

The study was approved by Hatfield NHS research ethics committee (13/EE/0357). Verbal consent was requested from healthcare professional and patients prior to the observations. Informed written consent was obtained prior to carrying out interviews. Interviews were audio recorded where interviewees also consented for recording; detailed notes were taken otherwise. Recordings were transcribed verbatim.

### Setting and participants

We studied two organisations, both large teaching hospital trusts in London. Organisation 1, based on one hospital site (430 beds), had used an in-house EP system since 2007. This was used on all inpatient wards with the exception of critical care areas; medication was prescribed on desk-based or tablet computers, or using computers-on-wheels. The same devices were also used by pharmacists to review inpatient medication orders and by nurses when administering medication. This site was purposively chosen to represent a hospital with an established inpatient EP system. Organisation 2 comprised five hospitals (total of 1,200 beds) and started introducing a commercially available EP system in a phased manner starting with two wards (elderly care and gynaecology) part way through the study, in March 2015. This site was chosen to give us the opportunity to study a paper-based inpatient prescribing system plus initial stages of roll out of a commercially available EP system. Both organisations had policies that allowed patients to self-administer their own medication if they wished and were assessed as competent to do so.

We studied a range of clinical areas with a focus on those admitting adults taking medication for long term conditions such as diabetes, Parkinson’s disease, HIV and chronic obstructive pulmonary disease. We included adult hospital inpatients, carers and healthcare professionals involved in their care. We excluded children, critical care areas and private healthcare.

### Study design

We conducted three phases of data collection across the two organisations. These comprised Organisation 1 (EP), Organisation 2 (pre EP), and Organisation 2 (post EP). We conducted ethnographic observations in each phase to observe how healthcare professionals facilitated patient understanding of their medication and patients’ involvement in medication safety, followed by semi structured interviews to explore patients’, carers’ and healthcare professionals’ views on inpatient involvement with their medication safety. We then triangulated the findings.

### Ethnographic study

Non–participant ethnographic observation was used to study the extent to which inpatients actively participated in medication safety activities, for example by providing information about their usual medication or by asking relevant questions, and how paper and electronic medication records were used (or not) by healthcare professionals to facilitate patient understanding of medication and to support patient involvement in medication safety. We observed nurses’ medication administration rounds, pharmacists’ ward rounds, doctor-led ward rounds and drug history taking for newly admitted patients by doctors and pharmacy staff. Sampling was purposive to include observations of EP at Organisation 1 and both paper prescribing and EP at Organisation 2. Detailed qualitative notes were made during the observations and typed up in full as soon as possible afterwards. Data collected included the location of the medication record and whether the medication record was shown to the patient as well as open notes of discussions about medicines, patient involvement in these, and any factors apparently affecting patient involvement. Verbal consent was requested from all healthcare professional and patients prior to the observations. At the end of the observations the researcher asked the healthcare professionals how they felt that being observed had affected them.

Three researchers (SG, SJ and AB) and four lay observers (FH, JL, CB and AT) conducted observations. The lay observers received training in research and information governance and were provided with the data collection forms that the researchers were using. The researchers co-observed some rounds together to confirm that they recorded similar information. The purpose of the lay observations was to obtain perspectives from lay people of different backgrounds. It was therefore not considered appropriate to train them to collect data from the same perspective as researchers nor test for reliability between the lay observers’ and the researchers’ observations [20]. However, we addressed validity by triangulating the data from observations with the interview data, searching for cases that contradicted the main findings and considering the extent to which the findings were supported by other relevant studies (cumulative validity). The majority of the observations were conducted by one researcher alone. However each of the lay observers co-observed a doctors’ ward round together with a researcher as well as observing a pharmacy round and some nurses’ drug administration rounds alone, with a researcher available on the ward if needed for support.

### Qualitative interviews

We conducted qualitative semi-structured interviews with healthcare professionals, patients and carers at each organisation. Semi-structured interview schedules were developed together with the study’s patient and clinical engagement group, and modified following the observations to explore the themes that arose, such as the reason for healthcare professionals holding some conversations about safety away from the patient. Interviews focused on respondents’ attitudes to patient involvement in preventing medication errors in the hospital setting, the use of the inpatient medication record in patient consultations, the perceived advantages and disadvantages of allowing patients to view their medication records, their opinion about involving patients in specific situations, such as informing them of errors, and (where relevant) any changes that had emerged as a result of the introduction of electronic records. We used an iterative process and building on early interviews we asked later interviewees about more specific scenarios. We subsequently asked about patients being informed about specific types of error: warfarin and lactulose overdoses, and errors that had not reached the patient, as it became apparent that healthcare professionals differed in their approach to informing patients about different types of errors. We adopted purposive sampling to include a wide range of different healthcare professionals, patients and carers. Healthcare professionals included doctors, nurses and pharmacists of different grades and gender, some of whom had been observed and some of whom had not. Patients with a range of long term conditions were recruited, including both genders, patients on short and long term medication and elective and emergency admissions. Patients were included if they were taking medication during their inpatient stay and were able to give informed consent. Nurses working on the relevant wards were asked to identify all eligible patients using these inclusion criteria. The researcher then selected patients using the purposive sampling strategy, provided information about the study and invited them to take part. Healthcare professionals on study wards were invited face to face or by email. Respondents were each interviewed once. Informed written consent was obtained prior to carrying out interviews. Interviews were audio recorded where interviewees also consented for recording; detailed notes were taken otherwise. Recordings were transcribed verbatim.

### Analysis

Interview transcripts and observation notes were coded using thematic analysis. Qualitative analysis software QSR NVivo was used to aid this process. The researcher started using a thematic open coding technique [21] but subsequently found that the codes identified showed considerable similarity to the contributory factors influencing safety in clinical practice proposed in the London Protocol (patient factors, task factors, individual healthcare professional factors, team factors, environmental factors and organisational factors) [22]. This framework was therefore adopted, but adapted to also incorporate ‘carer factors’ i.e. factors related to the patient’s carer; relevant subthemes were added to the contributory factors where relevant. One researcher, who was trained and experienced in qualitative analysis, coded all observation notes and interviews. Lay observers also wished to be involved in confirming that the London Protocol framework was appropriate. Therefore, a group of two researchers and four lay members received training in open coding and independently coded a sample of interviews using an open coding approach, without an *a priori* coding framework nor the use of NVivo. The sample of transcripts was purposively selected to include interviews with patients, carers, doctors, nurses and pharmacists. Any discrepancies among the group were resolved through discussion and any new themes were integrated into the framework. The interviews and observation coding were triangulated together in one NVivo file and the researcher searched for agreement and differences between these two data sets.

## Results

**Fig 1:**
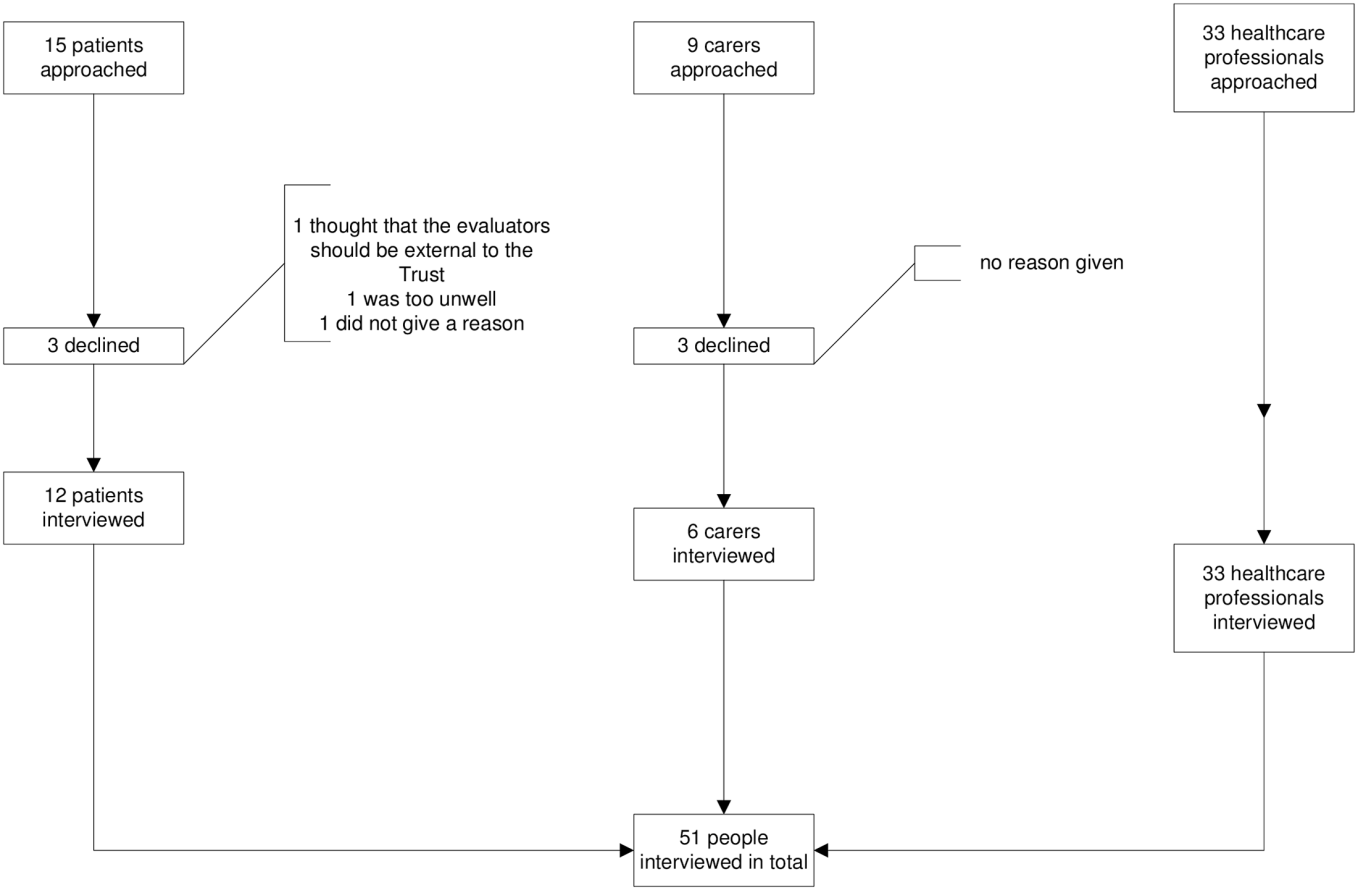
Flow chart of participants.

The number of patients observed during each type of round and the number of participants interviewed are shown in Table 1. Fifty one participants of 57 approached agreed to be interviewed, giving a response rate of 90% (Fig 1).

**Table 1:** summary of ethnographic observations and interviews conducted.

	Hospital organisation 1 (electronic prescribing)	Hospital organisation 2 (paper-based prescribing)	Hospital organisation 2 (electronic prescribing)	TOTAL
	**Observations**			
**Pharmacists’ ward rounds**	Researchers observed for *28 patients	Researchers observed for *66 patients	Researcher observed for 54 patients	**226 patients**
	**Lay observers observed for 0 patients	Lay observers observed for 24 patients	Lay observer observed for 54 patients	
**Doctors’ ward roundswithout pharmacist present**	Researchers observed visits to *14 patients	Researchers observed visits to *46 patients	Researcher observed visits to 36 patients	**132 patients**
	**Lay observers observed visits to 0 patients	Lay observer observed visits to 14 of these patients together with the researcher	Lay observer observed visits to 36 patients	
**Doctors’ ward rounds with pharmacist present**	Researchers observed visits to *21 patients	Researchers observed visits to *0 patients	Researchers observed visits to *0 patients	**21 patients**
	Lay observers observed visits to 4 of these patients together with the researcher	Lay observers observed visits to 0 patients together with the researcher	Researchers observed visits to *0 patients	
**Nurses’ drug administration rounds**	Researchers observed administration of medication to 30 patients	Researchers observed administration of medication to 33 patients	Researchers observed administration of medication to 32 patients	**130 patients**
	**Lay observers observed administration of medication to 0 patients	Lay observers observed administration of medication to 19 patients	Lay observers observed administration of medication to 16 patients	
**Prescribing/ drug history taking for new patients(no lay observations)**	Researchers observed for 15 patients	Researchers observed for 15 patients	***Researchers observed for 0 patients	**30 patients**
	**Interviews**			
Patients	4	4	4	12
Patients’ carers	2	2	2	6
Pharmacists	4	4	1****	9
Doctors	4	4	4	12
Nurses	4	4	4	12
Total	18	18	15	41

*At hospital organisation 1, pharmacists conducted some of their ward visits together with the doctors and some alone, but in hospital 2 pharmacists on the study wards conducted all their ward visits separately to the doctors.

** At hospital organisation 2 procedures for registering lay observers were more complex resulting in fewer lay observations at the site.

*** It was not possible to observe prescribing /drug history taking for new patients electronically on this site as this was still done on paper at the time of the study.

**** Only two pharmacists had experience of using electronic prescribing at the start of the study and only one was available for interview.

All interviews were recorded except for one with a carer and one with a nurse; detailed notes were taken for these. Interviews lasted between 10 and 45 minutes. No new codes were identified from the last few interviews and observation notes. Data saturation was therefore considered to have been reached by the end of data collection. The researcher and open coding group broadly identified the same themes.

Half of the observed healthcare professionals reported that being observed had not influenced their practice, with the others stating that they had felt nervous or had changed their practice in some way, such as increasing conversations about medicines with patients or spending more time looking at the drug chart.

Below we present the extent to which patients and health care professionals engaged in and reported supporting patient involvement in medication safety. We then present the facilitators and barriers to that involvement, structured according to our adaptation of the London Protocol [22]. Differences between electronic and paper based prescribing are described where they were observed or reported. However, while this affected patient involvement to some degree, many other factors emerged that appeared to influence patient participation.

### To what extent did patients and healthcare professionals perform and support patient medication safety behaviours?

#### How did healthcare professionals involve patients with their medicines during the observations?

During our observations, we saw that patient involvement with their inpatient medicines was often limited. We saw some instances of detailed information about medicines being given to patients, some where patients were asked for their opinions and were involved in decision making, and some where patients were asked if they had any questions about their medicines. However, we also observed many interprofessional conversations about medicines taking place away from patients or taking place in front of patients without involving them. For example, a consultant and pharmacist discussed which antibiotic to give a patient and decided to prescribe a cephalosporin even though the patient had a documented allergy to penicillin; the rationale was that the risk was small and the patient could be monitored while in hospital. We noted that the conversation between the consultant and pharmacist was very technical in nature; the patient was referred to in the third person and not asked about any known allergy to cephalosporins nor informed about signs of allergy to watch for. We also observed many interprofessional discussions where only a small fraction of the discussion between the healthcare professionals was relayed back to the patient e.g., ‘we’ll give you something for the swelling,’ [following decision by doctors to prescribe furosemide]. During nurse drug administration rounds, medication was not always prepared in the patient’s view and the patient was sometimes given a pot of medicines without being informed what they were. This limited opportunities for patient involvement in checking their medicines. The paper or electronic medication record was shown to patients in only 4 (2%) of 247 cases. However, medication records were sometimes used by healthcare professionals during discussions with patients and used to answer patients’ questions, without showing it to the patient. We did not observe any patients who were self-administering medication other than inhalers but subsequently interviewed one patient who was self-administering oral medication (see below).

#### Healthcare professionals’ views on patient involvement with their medicines

At the start of interviews, most healthcare professionals reported that they supported patient involvement in medication safety. However, it became clear during interviews that in many cases this support was somewhat limited. For example, some healthcare professionals reported supporting outpatient but not inpatient involvement, supporting giving patients some information but not informing them of all potential medication side effects so as to avoid anxiety, giving patients information but not involving them in decision making, or only involving them in practical decisions such as whether they would prefer a once or twice daily tablet. While all healthcare professionals were of the view that patients should be informed of any errors that led to significant harm, they differed as to exactly which other errors, if any, patients should be informed of. The majority of healthcare professionals thought that patients should be informed if their internationalised normal ratio (INR) had increased following a warfarin overdose, some thought they should be informed if they had diarrhoea following a lactulose overdose but only two healthcare professionals thought that patients should be informed of an error if the error had been corrected before it reached them. Most healthcare professionals reported that they supported patients viewing their medication records in theory but also that they did not actively encourage it in practice.

‘I’m always horrified when patients come and people say to me, ‘I’m on all these tablets, I don’t know what any of them are for’, and I say to them, ‘But, actually, it’s your body and you really ought to know what they’re for.’ (doctor 5 at start of interview; paper prescribing [PP])

‘I mean, there aren’t many patients who sit and study their [drug] charts, I always worry about the ones that do…. it usually indicates [laughs] a level of anxiety or obsession.’ (doctor 5 near end of interview; PP)

Some nurses did report occasionally showing patients their records. They stated that this was to reassure the patients that they were giving them the correct medication. Three healthcare professionals raised concerns about patients viewing their medication records such as patients’ misunderstanding or self-prescribing medication.

#### Patients’ reported preferences for involvement

In contrast, most inpatients initially reported that they did not think that they were involved in medication safety. However, it often emerged during the interviews that they were actually engaged in medication safety behaviours in practice such as checking the medication they had been given by the nurse.

Interviewer: ‘And would you want to be involved in checking that the right medicine is given in hospital?’

Respondent: ‘Not really, no, because… well I would hope they [healthcare professionals] do.’ (patient 2 early in interview; electronic prescribing [EP] organisation 1)

Respondent: ‘If something isn’t quite right I immediately say, “No, I’m not having that”, …It’s up to you to keep an eye on it… when I’m given it I’ll look to see what there is.’ (patient 2 later in interview; EP organisation 1)

Patients wanted to be informed about their medication and informed of any errors that had reached them, and to be able to take their medication at the same time they did at home. Interview data indicated that, like the healthcare professionals, most patients did not feel the need to be informed of errors that had been corrected before they reached them.

#### Differences between reported and observed behavior

Healthcare professionals reported involving patients in medication safety more than patients reported being involved and more than was observed in practice. For example one nurse reported that patients ‘should know’ what they are taking. However, the same nurse was observed handing patients tablets in a pot without explaining what they were. All healthcare professionals stated that they would support patients and carers expressing any concerns they had about medicines and they would respond to such concerns. However, some patients and carers reported that they had expressed concerns that had been ignored, and some healthcare professionals reported that other healthcare professionals may not respond to patient concerns. During the observations, patients raised 64 concerns about medicines, including adverse drug reactions, incorrect medication and dose omissions; 16 (25%) of these were not seen to be dealt with in any way. In other cases, healthcare professionals gave the patient information, took immediate action such as changing a prescription or drug administration or checking with other healthcare professionals, or promised that future action would be taken.

### Factors contributing to patient involvement in medication safety

#### Patient factors

**Health status:** Patients and healthcare professionals were of the view that factors relating to patients’ health status may make it more difficult for patients to be involved with medication safety while in hospital. On admission, patients were likely to be acutely unwell and not always fully conscious. One healthcare professional described the negative emotions that patients may go through during their hospital stay, such as anxiety about test results and feelings of depression on receipt of bad news. This healthcare professional was of the view that correct timing of discussions about medication with patients may be critical to optimise patient involvement. A patient described how difficult it was to be assertive when unwell, particularly if concerns were not addressed when expressed the first time.

**Physical and medical needs:** In addition, interview data demonstrated that specific physical or medical issues may need to be considered in order to optimise patient involvement in medication safety. These included lack of mobility to access medication records kept at the end of their bed, visual impairment making it difficult to read written information, hearing impairment, speech impairment, cognitive impairments and psychiatric illness. For example, one pharmacist suggested that having a hearing aid battery service on hospital wards would aid communication.

**Patients’ knowledge and beliefs:** Both healthcare professionals and patients identified that patients’ knowledge and beliefs could affect their involvement. Both groups thought that some patients were more knowledgeable and interested in their medication than others and that some were more assertive and others more passive. Some patients described having ‘blind faith’ in healthcare professionals to manage their medication and did not think that their involvement was necessary. Other patients expressed concern that they may upset healthcare professionals and that their care would be affected if they challenged healthcare professionals.

‘As a patient, you have to go along with the system, and if you don’t you might as well… well, might as well discharge yourself really because you get the backs up—presumably, I’ve never tried this—but you get a lot of unhappy people, wouldn’t you? Sooner or later you get a reputation for being uncooperative and I definitely wouldn’t like that because you wouldn’t get the best possible treatment then. ‘(patient 6, PP)

Healthcare professionals reported an awareness of these patient concerns and some were of the view that older patients may be more likely to hold these beliefs. Interview data demonstrated that many patients did not know what was termed by one of our lay observers the “rules of engagement”. They were not aware of the possibility of medication self-administration or their rights to view their own medication record. One patient reported that he had asked for self-administration in another hospital and told it was not allowed and as a result had not enquired about it on this occasion. There was also evidence from the interviews that some patients did not know the roles of different healthcare professionals or who to ask if they had questions, although others appeared to have a clearer understanding of different roles.

#### Carer factors

**Carer role:** Carers and healthcare professionals identified that a carer could have an important role as an advocate for inpatients’ medication safety and we saw examples of this in our observations. However, interview data also showed that while carers had an essential role in medicines management in the community setting, this involvement was greatly reduced when patients came into hospital; some carers viewed this as undesirable. On the other hand, one healthcare professional expressed the view that carers could sometimes take responsibility away from the patient, so that the patient themselves became less involved with their own medication safety. During the observations we observed some three-way conversations between patients, carers and healthcare professionals but also some where the interaction was between the carer and healthcare professional only, excluding the patient.

Healthcare professionals reported that informal carers who were relatives of the patient were more likely to be involved than formal agency carers.

‘Carers from agencies potentially don’t usually visit the patient that often.’ (pharmacist 9 [EP] Organisation 2).

**Carers’ knowledge and beliefs:** It became apparent from the analysis that carers’ beliefs about their role and their knowledge of the ‘rules of engagement’ also affected how involved they were in the medication safety of the people they normally cared for during their inpatient stay. Like some patients, some carers believed that medication was the domain of the healthcare professionals and that it was not their role to be involved. It may be more difficult for carers to get involved as they do not have a right to view the patients’ medication records without the patients’ explicit consent, an issue specifically mentioned by some healthcare professionals.

#### Task Factors

**Paper versus electronic prescribing:** Interview data suggested that EP could be a specific barrier to patients accessing their medication records. While some patients and carers reported viewing their paper based medication records, none had viewed EP records. One patient reported that he had asked to view his EP medication record but this had not happened. Healthcare professionals at that hospital explained that it was not possible to give patients access to their medication record on an unsupervised individual basis, as the software gave healthcare professionals password-protected access to all patient medication records.

‘On the [name of EP system] you don’t just have that one patient, there’s everyone’s patients’ details on there so you couldn’t just give a patient a computer with their drugs on and be like, “Have a look,” because they could go onto anything.’ (Nurse 2 EP Organisation 1)

Healthcare professionals also reported that there was no ‘patient friendly’ interface for viewing medication records and some were of the view that the electronic record was less comprehensible to the patient than the paper drug chart.

At Organisation 2, we also observed confusion caused by the use of paper based prescribing on some wards but EP on others due to the phased EP roll out. This led to dual drug charts and a carer being confused about the stop date of her relative’s medication. The stop date that she had viewed on the paper chart was not the actual stop date but the date the medication records had been transferred to EP.

**Medication-related factors:** Some aspects of a medication regimen were perceived to affect inpatient involvement. Some healthcare professionals reported that they would be less likely to involve inpatients if the medication regimen was unstable and constantly changing while they were in hospital. Conversely, interview and observation data suggested that patients taking long term antiretrovirals or medication for Parkinson’s disease were more likely to be involved with their medication safety in hospital than other patients. During drug administration rounds, nurses were generally observed discussing ‘when required’ medication with patients more often than regular medication.

Patients, healthcare professionals and carers all identified that patients may find it more difficult to be involved in their medication in hospital than in the community because of unfamiliar formulations of drugs, such as intravenous preparations. They also identified that the appearance of medication from different manufacturers may differ, which may make it more difficult for patients to identify their medication. During nurses’ drug administration rounds, nurses were observed informing patients of the medication being given orally more often than medication being given intravenously or via a nebuliser. Patients were more likely to maintain control of, and self-administer, inhalers than other formulations.

**Stage of hospital stay:** Interview and observation data suggested that healthcare professionals focused more on taking a drug history from patients on admission and preparing them for medication regimens on discharge, than involving them with their medication safety as inpatients in between admission and discharge. In observations, healthcare professionals always included the patient in confirming a drug history on admission. When healthcare professionals were asked about involving inpatients in their medication safety, many discussed the importance of the patient as a source of information at admission, particularly out of office hours, and the importance of making sure they could manage their medication after discharge.

#### Individual healthcare professional factors

Healthcare professionals expressed a range of beliefs about patient involvement, with some being more encouraging of involvement than others. Interview data suggested that healthcare professionals facilitated involvement if they were confident and secure in allowing patients to express their concerns, if they were able to imagine themselves in the role of the patient and if they identified that patients have a great deal of knowledge of their own medication and should be listened to. They were observed facilitating this involvement by actively engaging with patients and showing a willingness to listen. One pharmacist reported that another pharmacist had given her work contact number to patients in case they had concerns about their medication while in hospital. Another pharmacist thought that patients may be more likely to express concerns to pharmacists and allied healthcare professionals who were not as directly involved with their care, rather than to doctors and nurses.

However, on the other hand some healthcare professionals expressed fears and concerns that prevented them from facilitating patient involvement with medication safety. These included fears of being sued if they informed patients of errors, fears that they would not be able to answer patients’ questions, concern that patients may misinterpret information or alter drug charts, burdening patients with too much information, and worrying anxious patients about medication side effects. Some of these fears were based on the individuals’ reported experiences of not being able to answer questions or a patient’s misinterpretation of information written on the drug chart, which caused the patient to become very worried. However, other healthcare professionals’ fears seemed more theoretical. While some healthcare professionals were of the view that they may face litigation if they informed patients of errors, one patient reported that she would sue healthcare professionals if they did NOT keep her informed of errors and one healthcare professional recognised that being open and honest about errors made patients more accepting as well as being the right thing to do.

‘I wouldn’t never [ever] tell a patient directly an error has been made, because that can be very alarming for the patient, and then can lead to potential law suits and things like that. (pharmacist; 8 PP)

Interviewer: ‘Is that something you would want to be told about?’

Respondent: ‘I’d sue them if they didn’t.’

Interviewer: ‘if they didn’t inform you of what was going on?’

Respondent: Yes.(patient 7; PP)

These findings suggest that healthcare professionals’ attempts to reduce litigation by concealing errors are likely to be counterproductive.

#### Team factors

**Team dynamics:** The way in which healthcare professionals operated as a team appeared to act as a barrier to patient involvement. A medication error or concern was often identified by a different healthcare professional to the one originally involved, making team dynamics important. In the observations, many conversations where one healthcare professional was challenging another were held away from the patient. In the interviews, when healthcare professionals were asked how comfortable they would be involving patients in these discussions, the vast majority reported that they would prefer to hold them away from patients, due to concern about presenting an uncertain and worrying picture to patients as well as concerns about undermining their colleagues. One pharmacist explicitly stated that a consultant would be less likely to take his advice if he challenged her in front of a patient.

#### Whose responsibility is patient involvement?

Another issue that arose was that while all healthcare professionals thought patient involvement in medication safety was a good idea, many thought that another healthcare professional group should be ones the doing it. This suggests that any solutions would have to take account of multidisciplinary working.

‘Maybe just more a nurse engagement with their medicines … just to discuss, “This is your medication … and this is what it’s for, this is why you’re taking it,” actually at the point of actually giving it, and maybe doctors discussing it a bit more when they make changes to medicines.’ (pharmacist 2; EP Organisation 1)

‘I think it’s good that we have pharmacists because they can definitely help with that, but doctors on a ward round… there’s lots of patients to see and time constraints.’ (doctor 4; EP Organisation 1)

‘We then kind of push a bit of the responsibility on to the nurses; so they think it’s our responsibility, we think it’s their responsibility and it kind of falls between the gap.’ (pharmacist 5; PP)

#### Who has the knowledge to involve patients?

A related issue was that doctors, pharmacists and nurses all have their own roles and specialities concerning medicines, which means that no one healthcare professional may have the full knowledge to be able to answer all patients’ questions. Nurses reported that they did not have in depth knowledge about the medication they were administering or the reasons for prescribing. Doctors stated that they were able to tell the patients the name of the medication they have been prescribed and why, but were unable to relate it back to ‘that small white tablet she [the nurse] gave me this morning.’

#### Environmental factors

**Location of medication record:** Observational data suggested that the location of the medication record affected patient involvement. When medication records were located or available at the patient’s bedside, conversations about medicines seemed more likely to be held in front of patients. In addition, healthcare professionals accessed these records to inform patients about their medication. However, where the medication record was unavailable, either because a paper-based drug chart was not at the end of the bed, or a portable computer on wheels was not available to view electronic records, healthcare professionals were sometimes unable to answer patients’ questions.

These findings were supported by healthcare professional interview data.

‘I think the problem with electronic prescribing is that you often don’t have the drugs chart there at the bedside with the patient so often what will happen is we’ll go through everything on a computer before we go in then we go and see the patient, and it’s not often, you know, our patients are on 10 drugs, 15 drugs, and you forget what they’re on, and you can’t go through that with the patient at the bedside. So I think that kind of makes it difficult sometimes.’ (doctor 2; EP Organisation 1)

Interview and observation data demonstrated that the layout of the ward also affected whether electronic medication records were available at the patient bedside. In Organisation 1 where EP was well established, the computers on wheels could be wheeled to the head of the patient’s bed but on the ward in Organisation 2 there was insufficient space.

‘So unfortunately some of the bed spaces are quite narrow so say the patient’s got their legs up on the edge of the table it’s quite hard to really roll it around to them so they can have a look. ‘ (doctor 10; EP Organisation 2)

In addition where there were no electrical sockets near a patient’s bedside, computers on wheels were not available if they were not already charged.

**Other environmental factors:** In addition, the busyness of the ward environment was seen by patients and healthcare professionals as a barrier to patient engagement. Finally, observation data demonstrated that where patients or medications were subject to infection control precautions, this often worked against patient engagement. Some medication, such as intravenous medication, was prepared in a dedicated area, usually a treatment room where equipment and medication are stored. Where patients were isolated in a side room for infection control purposes, the medication record and medication storage were likely to be located outside the room.

#### Organisational factors

It became apparent from the analysis that the policies and messages given to healthcare professionals about patient involvement seemed to be contradictory. On the one hand, healthcare professionals were encouraged to be open and honest with patients, keep them informed. and involve them in decision making. However, on the other hand, the rigid and formal structures of the hospital could hinder patient involvement. For example, nurses are required to administer medication exactly according to the doctor’s prescription and interview data demonstrated that some nurses were reluctant to deviate from these instructions even when patients raised safety concerns. One nurse reported that another nurse had refused to adjust a patient’s insulin in accordance with the way the patient reported she used insulin at home, as that deviated from the prescription, leading to the patient having a potentially avoidable hypoglycaemic episode. Another patient, who was self-administering most of his oral medication, was not allowed to self-administer his controlled drug, meaning that he was not able to take it at the time he wanted to. Drug administration rounds, doctors’ ward rounds and pharmacy visits were scheduled to take place at specific times and these were not always the times that were optimal for patient and carer involvement. For example, the vast majority of doctors’ ward rounds and pharmacy visits took place outside of visiting hours, which meant that carers were rarely present. Both hospitals provided some flexibility in allowing the ‘main carer’ to be present outside of visiting hours. However the researchers observed that there was some uncertainty among healthcare professionals about carers being there outside visiting hours, although when they were present the researchers observed them giving information and assistance to the healthcare professionals. When carers were not present, junior healthcare professionals reported that they had to try and contact them at a later time to gain information; in other cases this led to a lack of carer involvement.

Interviewer: ‘So what sort of opportunities are there for interactions with carers on the ward?’

Respondent: ‘Mostly serendipitous, just by virtue of them being there during ward rounds or potentially drug rounds as well. ‘ (doctor 9; EP Organisation 2)

Healthcare professionals, patients and carers expressed the view that there was a general institutional culture in hospitals where patients were expected to take on the ‘sick role’ and allow the healthcare professionals to ‘get on with their job’.

The more elderly patients, some are very involved with their medication, but a lot just let it lapse and just get quite used to the hospitalisation because especially if they’re in a for a long stay they’re used to somebody else doing everything for them.(pharmacist 2; EP)

We patronise people, we treat people like little babies, you know (nurse 8; PP)

Well I don’t think patients are particularly involved at the moment because it’s all based on discussion between inter-professionals and you don’t really get a choice in what happens to you once you’re in a bed in the hospital. You’re given the medication and you don’t see what you’re being given, especially if it’s given to you in a little unnamed pot. (pharmacist 3; EP Organisation 1)

I don’t think they know that listening is part of the job. I think it’s very much a culture of we tell you what to do whilst you’re here (patient 7; PP).

## Discussion

The study findings build on previous research that has identified the challenges that may be involved in more active engaging inpatients with medication safety. Previous research [6,14,23–27] and the current study have all identified that patients’ knowledge, and patients’ and healthcare professionals’ beliefs are an important factors in the level of patient involvement in the inpatient setting. In addition, both previous research [25–26] and the present study have identified that the different formulations of medication in hospital may make it more difficult for patients to be involved, giving these findings cumulative validity. The limited opportunities for carers to communicate with healthcare professionals about patients’ medicines has been reported in Australia [28] and the current study has found this also to be an issue in the UK.

The current study has also identified the importance of considering team dynamics and factors related to the hospital environment and organisational structure when developing interventions to increase patient engagement with medication safety. Healthcare professionals in hospital do not work in isolation but as part of a multidisciplinary team. The dynamics within the team need to leave space for the patient to become involved. While there is currently a political drive to increase patient involvement in hospitals, there are other potentially competing priorities, such as infection control and rigorous procedures for medication use. Therefore there may be a conflict between decreasing medication errors by increasing patient engagement and other protocols designed to increase safety for inpatients.

Paper based versus electronic prescribing seemed to have some effect on patients’ access to medication records as electronic records could only be accessed via a healthcare professional, whereas paper based records were often potentially available to patients at the end of their bed. Similarly, a mixed-methods study using multichannel video recording and in-depth interviews in UK primary care found that none of the available electronic systems consider or accommodate the possibility that patients may ‘share the screen’ with the healthcare professional [29]. In the UK inpatient setting, Lee et al [30] found that the computer screen was virtually invisible to inpatients and that the lack of a physical presence of a drug chart could prevent patients being involved with their medication.

However, the way in which healthcare professionals share and use medication records within patient consultations was also an important factor. Milne et al [29] identified three ways in which healthcare professionals share healthcare records with patients: convincing, translating and verifying. In the current study, on the rare occasions where the medication record was shared with patients during consultations, this was done for the purpose of “convincing” patients to take the medication prescribed by the doctors, rather than helping patients and healthcare professionals reach a shared understanding (translating) or enabling patients to question the medication they had been prescribed (verifying). Patients were not routinely shown their medication record. However, sharing the record for the purpose of verification has been found to improve patient safety in primary care, primarily through patients identifying errors in medication lists and adverse drug reactions [31]. Lee et al [30] have also suggested that there needs to be an underlying cultural change in how the patient’s role is perceived by clinicians in order for electronic prescribing to be used as a tool to improve communication with patients.

### Strengths and Limitations

Strengths of our study are that data were collected from a large number of different stakeholders, and that we triangulated observation and interview data to give an overall picture of current inpatient involvement in medication safety and the challenges to consider when developing interventions to increase this. Lay members were involved in all stages of the project alongside researchers, including data collection and analysis.

The observer was reported to have had some effect on the healthcare professionals observed in half the cases. The actual engagement of patients may therefore have been slightly higher than usual practice.

The study took place in two purposefully selected hospital organisations, and these were both within the same geographic area in the UK. It is unclear how generalisable these findings would be across the UK or internationally. However, the study has identified important areas to consider when developing interventions to increase patient involvement in medication safety in any setting. Future research should focus on the development of such interventions.

## Conclusions

This study had identified that patient involvement in medication safety is often limited in the inpatient hospital setting. The findings suggest that to develop interventions to increase patient engagement with medication safety behaviours, a wide range of factors need to be considered, including those relating to the hospital environment, health care team and organisational culture, as well as those relating to individual patients, healthcare professionals and tasks.

## Abbreviations

EP: electronic prescribing
PP: paper based prescribing
